# Insulin resistance induces earlier initiation of cognitive dysfunction mediated by cholinergic deregulation in a mouse model of Alzheimer's disease

**DOI:** 10.1111/acel.13994

**Published:** 2023-10-11

**Authors:** Naotaka Izuo, Nobuhiro Watanabe, Yoshihiro Noda, Takashi Saito, Takaomi C. Saido, Koutaro Yokote, Harumi Hotta, Takahiko Shimizu

**Affiliations:** ^1^ Department of Endocrinology, Hematology and Gerontology, Graduate School of Medicine Chiba University Chiba Japan; ^2^ Department of Pharmaceutical Therapy and Neuropharmacology, Graduate School of Medical and Pharmaceutical Sciences University of Toyama Toyama Japan; ^3^ Department of Autonomic Neuroscience Tokyo Metropolitan Institute for Geriatrics and Gerontology Tokyo Japan; ^4^ Department of Animal Facility Tokyo Metropolitan Institute for Geriatrics and Gerontology Tokyo Japan; ^5^ Laboratory for Proteolytic Neuroscience RIKEN Center for Brain Science Wako Japan; ^6^ Department of Neurocognitive Science Institute of Brain Science, Nagoya City University Graduate School of Medical Sciences Nagoya Japan; ^7^ Aging Stress Response Research Project Team National Center for Geriatrics and Gerontology Obu Japan

**Keywords:** Alzheimer's disease, cerebral blood flow, cholinergic system, cognitive dysfunction, insulin resistance, nicotinic acetylcholine receptor α7

## Abstract

Although insulin resistance increases the risk of Alzheimer's disease (AD), the mechanisms remain unclear, partly because no animal model exhibits the insulin‐resistant phenotype without persistent hyperglycemia. Here we established an AD model with whole‐body insulin resistance without persistent hyperglycemia (APP/IR‐dKI mice) by crossbreeding constitutive knock‐in mice with P1195L‐mutated insulin receptor (IR‐KI mice) and those with mutated amyloid precursor protein (*App* ^*NL‐G‐F*^ mice: APP‐KI mice). APP/IR‐dKI mice exhibited cognitive impairment at an earlier age than APP‐KI mice. Since cholinergic dysfunction is a major characteristic of AD, pharmacological interventions on the cholinergic system were performed to investigate the mechanism. Antagonism to a nicotinic acetylcholine receptor α7 (nAChRα7) suppressed cognitive function and cortical blood flow (CBF) response to cholinergic‐regulated peripheral stimulation in APP‐KI mice but not APP/IR‐dKI mice. Cortical expression of *Chrna7*, encoding nAChRα7, was downregulated in APP/IR‐dKI mice compared with APP‐KI. Amyloid β burden did not differ between APP‐KI and APP/IR‐dKI mice. Therefore, insulin resistance, not persistent hyperglycemia, induces the earlier onset of cognitive dysfunction and CBF deregulation mediated by nAChRα7 downregulation. Our mouse model will help clarify the association between type 2 diabetes mellitus and AD.

## Graphical abstract

Although insulin resistance increases the risk of Alzheimer's disease (AD), the mechanisms remain unclear, partly because no animal model exhibits insulin resistance without persistent hyperglycemia. AD model mice with constitutive knock‐in of mutant insulin receptors, which developed insulin resistance without persistent hyperglycemia, exhibit earlier cognitive dysfunction with cholinergic deregulation. Our mouse model will help clarify the association between type 2 diabetes mellitus and AD.

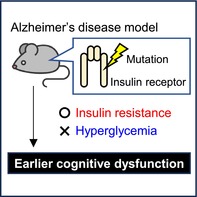

**Abbreviations**
AD: Alzheimer’s disease
APP: amyloid precursor protein
Aβ: amyloid β
CBF: cortical blood flow
CNS: central nervous system
ELISA: enzyme‐linked immunosorbent assay
GTT: glucose tolerance test
IGF: insulin‐like growth factor
IR: insulin receptor
ITT: insulin tolerance test
nAChRα7: nicotinic acetylcholine receptor α7
NBM: nucleus basalis of Meyne
PBS: phosphate‐buffered saline
RT‐PCR: real‐time reverse transcription polymerase chain reaction
SEM: standard error of the mean
T2DM: type 2 diabetes mellitus
TBS: tris‐buffered saline
WT: wild‐type

## 1 INTRODUCTION

Alzheimer's disease (AD) is a neurodegenerative disease characterized by memory, cognition, and reasoning deficits. Senile plaques are commonly observed as insoluble deposits in AD patients (Haass & Selkoe, 2007) that had begun to accumulate in the brain long before diagnosis (Bateman et al., 2012). Risk factors for AD (e.g., metabolic syndrome, psychological stress, and dietary patterns) may accelerate disease pathology in the preclinical stage and induce an earlier onset of clinical symptoms (Edwards et al., 2019; Livingston et al., 2020). Epidemiologically, the manifestation of type 2 diabetes mellitus (T2DM) in middle age has been reported to double the risk of AD (Biessels et al., 2006; Ott et al., 1999; Zuin et al., 2021). Considering that senile plaque deposition begins 20 years before the diagnosis (Bateman et al., 2012), T2DM may modify the underlying pathology in the preclinical stage of AD.

Among the clinical parameters of T2DM, glucose intolerance and acute glucose fluctuation, but not fasting glucose levels, are correlated with cognitive decline (Ohara et al., 2011; Rizzo et al., 2010; Zhong et al., 2012). These studies suggest that insulin resistance is the main contributor to AD exacerbations induced by T2DM. The mechanisms of AD exacerbation have been investigated using conventional rodent models established through high‐fat diet feeding (Amelianchik et al., 2021; Martins et al., 2017) or peripheral streptozotocin administration (Zangerolamo et al., 2021). These models suppress systemic insulin signaling accompanied by severe hyperlipidemia or hyperglycemia, possibly leading to misunderstandings about the effect of downregulated insulin signaling. In this study, we used knock‐in mice with a mutation (P1195L) in the insulin receptor (IR) (IR‐KI mice) (Baba et al., 2005). IR‐KI mice exhibit insulin resistance without persistent hyperglycemia throughout life (Baba et al., 2005; Shimizu et al., 2011). Furthermore, we selected knock‐in mice with KM670/671NL, E693G, and I716F mutations in the amyloid precursor protein (*App* ^*NL‐G‐F*^ mice; APP‐KI mice) as a model for the preclinical stage of AD, which exhibits severe accumulation of amyloid β (Aβ) (Saito et al., 2014; Sasaguri et al., 2017), a major component of senile plaques, in the mouse brain. We crossbred the IR‐KI and APP‐KI mice and analyzed the resultant mice (APP/IR‐dKI mice) to investigate the pathological role of insulin resistance in AD exacerbation.

Acetylcholine is an essential neurotransmitter for various functions of the central nervous system, such as attention, memory, motivation, and arousal. Cholinergic projections from the nucleus basalis of Meynert (NBM) to the cerebral cortex are involved in cognitive function and blood flow control. Direct electrical stimulation of the NBM induces a region‐wide increase in cortical blood flow (CBF) (Biesold et al., 1989; Hotta, 2016; Kurosawa et al., 1989; Zhang et al., 1995). Peripheral sensory input also activates the NBM and increases CBF mediated by acetylcholine and its muscarinic and nicotinic receptors (Akaishi et al., 1990; Kurosawa et al., 1992; Uchida et al., 2000). In addition to cholinergic dysfunction of the projections, neuronal loss in the NBM has been observed in patients with AD (Davies & Maloney, 1976; Nordberg et al., 1992; Schliebs & Arendt, 2011; Whitehouse et al., 1982). Elevation of presynaptic acetylcholine levels by the pharmacological inhibition of its degrading enzyme has beneficial effects on cognition and behavior in patients with AD. Interestingly, the expressions of the cholinergic receptors were decreased in the hippocampus of autopsy brains of patients with T2DM and its model mice (Xu et al., 2020). Therefore, the involvement of cholinergic alterations in the modified pathology of patients with AD affected by T2DM has been speculated.

In the present study, to clarify the effects of insulin resistance on the preclinical pathology of AD, we compared APP‐KI mice with APP/IR‐dKI mice by focusing on cholinergic function. APP/IR‐dKI mice, which exhibited insulin resistance without persistent hyperglycemia, showed early initiation of cognitive dysfunction and altered CBF regulation mediated by downregulation of the nicotinic acetylcholine α7 receptor (nAChRα7). Analyses of APP/IR‐dKI, whose cognitive signature could simulate that of AD patients with T2DM, suggest that AD exacerbation by insulin resistance is mediated by impaired nAChRα7.

## 2 RESULTS

### 2.1 APP/IR‐dKI mice exhibit insulin resistance and abnormal blood glucose fluctuation but not persistent hyperglycemia

**FIGURE 1:**
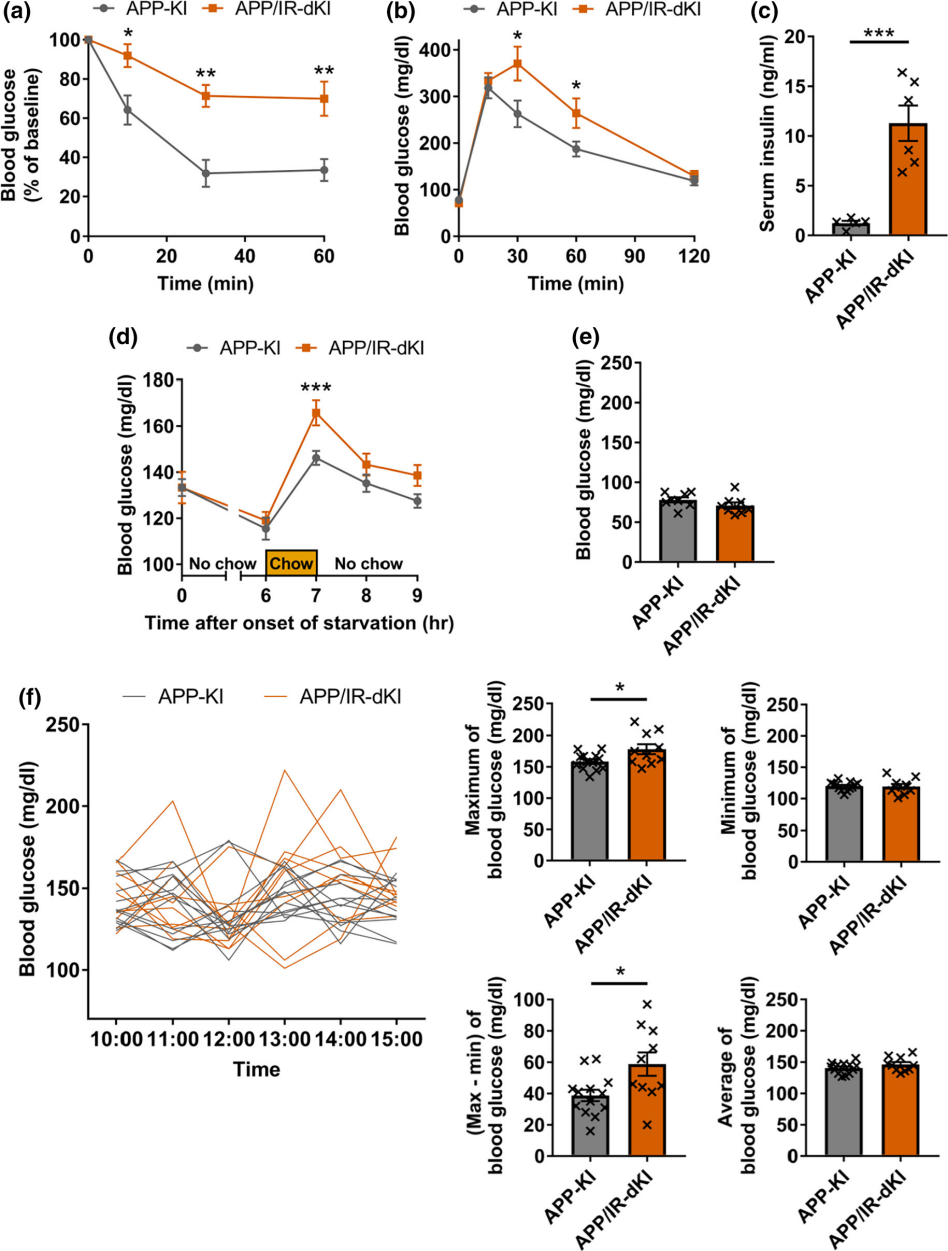
APP/IR‐dKI mice exhibit insulin resistance and abnormal glucose fluctuations but not persistent hyperglycemia at 3 months of age. (a) Insulin intolerance was observed in APP/IR‐dKI mice after insulin administration (APP‐KI mice, *n* = 8; APP/IR‐dKI mice, *n* = 6). (b) Glucose intolerance was observed in APP/IR‐dKI mice after glucose administration (APP‐KI mice, *n* = 8; APP/IR‐dKI mice, *n* = 8). (c) Higher serum insulin levels at fed state were observed in APP/IR‐dKI mice than in APP‐KI mice (APP‐KI mice, *n* = 5; APP/IR‐dKI mice, *n* = 6). (d) Higher glucose elevation following chow consumption was observed in APP/IR‐dKI mice than in APP‐KI mice (APP‐KI mice, *n* = 13; APP/IR‐dKI mice, *n* = 10). (e) No difference was observed in fasting glucose levels between APP‐KI mice (*n* = 8) and APP/IR‐dKI mice (*n* = 8). These data correspond to 0 min in graph (b). (f) Blood glucose was serially monitored every 1 h for 6 h (APP‐KI mice, *n* = 13; APP/IR‐dKI mice, *n* = 10). Higher maximum levels, but not higher minimum levels, were observed in APP/IR‐dKI mice than in APP‐KI mice. The gap between the maximum and minimum levels over 6 h, but not the average level, was also elevated in APP/IR‐dKI mice. Values represent the mean ± SEM *p < 0.05, **p < 0.01, ***p < 0.001.

APP‐KI mice exhibit Aβ deposition at 2 months of age and cognitive impairment at 6 months of age (Saito et al., 2014). In the present study, systemic glucose metabolism was examined at 3 months of age, and other analyses, including behavioral analyses, were performed at 4 months of age. Since female IR‐KI mice do not exhibit insulin resistance because of estrogen (Baba et al., 2005), male mice were chosen in all of the experiments in this study. APP‐KI mice exhibited normal glucose metabolism (Figure S1). APP/IR‐dKI mice exhibited typical features of insulin resistance in the insulin tolerance test (ITT) (*p* = 0.0174 at 10 min, *p* = 0.0047 at 30 min, and *p* = 0.0033 at 60 min; Figure 1a) and glucose tolerance test (GTT) (*p* = 0.182 at 0 min, *p* = 0.612 at 15 min, *p* = 0.0350 at 30 min, *p* = 0.0281 at 60 min, and *p* = 0.293 at 120 min; Figure 1b) and higher serum insulin levels compared with APP‐KI mice (*p* = 0.0007; Figure 1c). Chow consumption after starvation for 6 h also increased blood glucose levels in APP/IR‐dKI mice (*p* = 0.999 at 0 h, *p* = 0.583 at 6 h, *p* = 0.0035 at 7 h, *p* = 0.194 at 8 h, and *p* = 0.0936 at 9 h; Figure 1d). APP/IR‐dKI mice exhibited normal blood glucose levels after overnight starvation (*p* = 0.182; Figure 1e), probably because higher levels of circulating insulin compensated for downregulated insulin signaling in their tissues. To investigate the regulation of blood glucose levels under normal conditions, blood glucose levels were serially monitored every hour at six time points (Figure 1f). At each time point, higher maximum levels (*p* = 0.0263), but not minimum levels (*p* = 0.909), were observed in APP/IR‐dKI mice than in APP‐KI mice. Significant differences in the gap between the maximum and minimum levels of the two groups were also observed (*p* = 0.0174). Similar to the fasting glucose levels, there was no difference in the average levels at all time points between the groups (*p* = 0.170). Taken together, APP/IR‐dKI mice exhibited insulin resistance and larger glucose fluctuations than APP‐KI mice did, without persistent hyperglycemia. Thus, APP/IR‐dKI mice were burdened with the pathological components of T2DM related to the exacerbation of AD, as revealed in epidemiological and clinical studies (Biessels et al., 2006; Ott et al., 1999; Zuin et al., 2021).

### 2.2 APP/IR‐dKI mice exhibit cognitive dysfunction related to nicotinic acetylcholine alpha 7 receptor signaling at the earlier age

**FIGURE 2:**
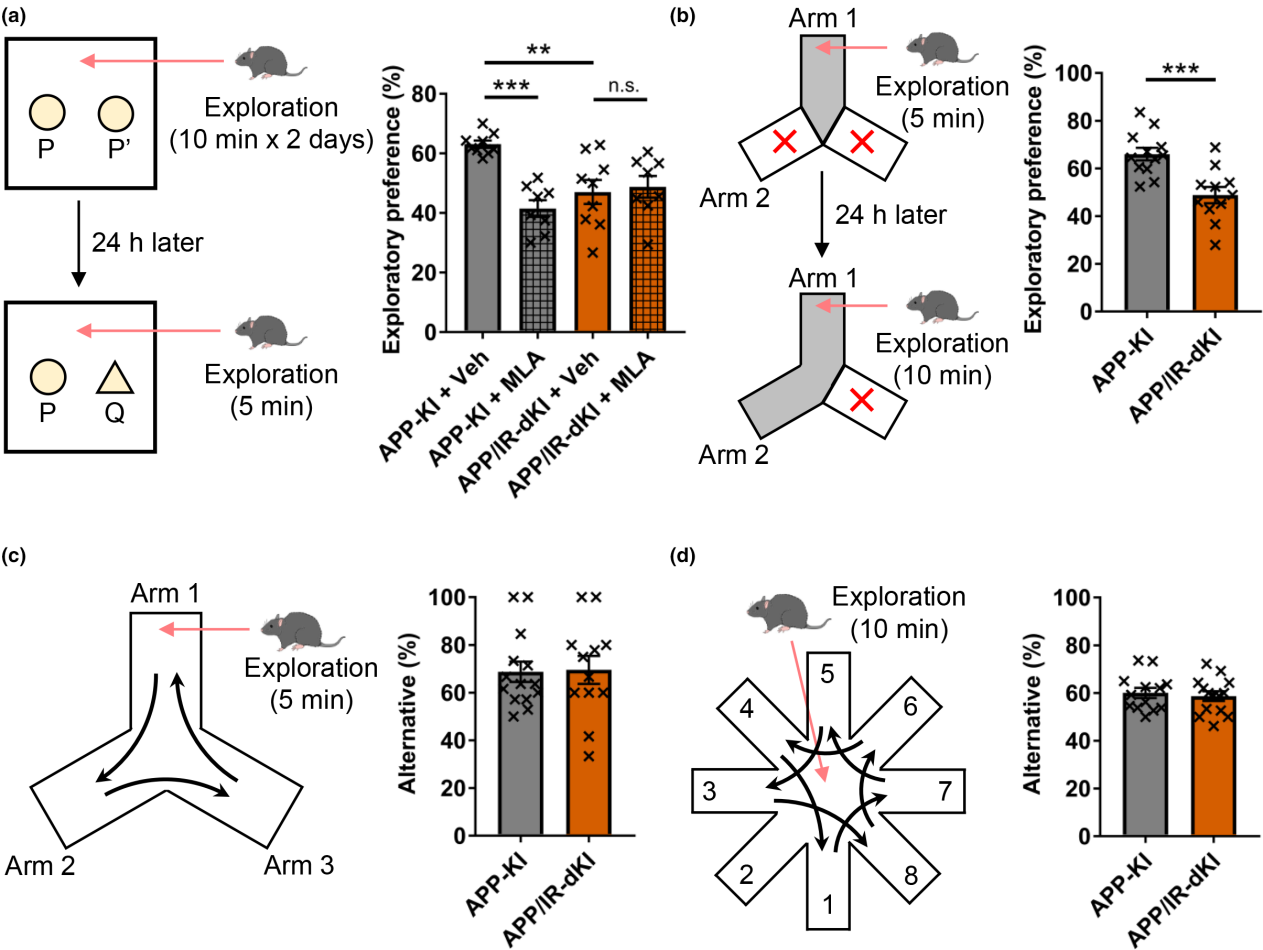
APP/IR‐dKI mice exhibit cognitive dysfunction at 4 months of age. (a) In the novel object recognition test, APP/IR‐dKI mice exhibit decreased exploratory preference compared with APP‐KI mice. MLA administration at 15 min before the test lowered the exploratory preference in APP‐KI mice but not in APP/IR‐dKI mice (APP‐KI mice + vehicle (Veh), *n* = 9; APP‐KI mice + MLA, *n* = 8; APP/IR‐dKI mice + Veh, *n* = 9; APP/IR‐dKI mice + MLA, *n* = 8). (b) In the novel place preference test, APP/IR‐dKI mice exhibited lower exploratory preference compared with APP‐KI mice (APP‐KI mice, *n* = 12; APP/IR‐dKI mice, *n* = 11). (c and d) No differences in alternation were found in the three‐arm or eight‐arm maze tests between APP‐KI and APP/IR‐dKI mice (three‐arm maze test: APP‐KI mice (*n* = 14) and APP/IR‐dKI mice (*n* = 12); eight‐arm maze test: APP‐KI mice (*n* = 13) and APP/IR‐dKI mice (n = 14)). Values represent the mean ± SEM **p < 0.01, ***p < 0.001.

The cognitive functions of APP/IR‐dKI mice were evaluated using behavioral tests at the age of 4 months, when severe cognitive dysfunction does not emerge in APP‐KI mice (Masuda et al., 2016; Saito et al., 2014). A novel object recognition test was conducted to evaluate long‐term memory. APP/IR‐dKI mice showed significantly lower discrimination of novel objects than APP‐KI mice did. Although administration of the nAChRα7 antagonist MLA shortened the exploration time of a novel object taken by APP‐KI mice, a further inhibitory effect on memory was not observed upon MLA administration in APP/IR‐dKI mice (APP‐KI mice + vehicle (Veh) vs. APP/IR‐dKI mice + Veh, *p* = 0.0046; APP‐KI mice + Veh vs. APP‐KI mice + MLA, *p* = 0.0002; APP/IR‐dKI mice + Veh vs. APP/IR‐dKI mice + MLA, *p* > 0.9999; Figure 2a). These results suggest that the long‐term memory of APP/IR‐dKI mice was impaired by nAChRα7 dysfunction. In the novel place preference test, APP/IR‐dKI mice were confirmed to have decreased exploratory preference for a novel place compared with APP‐KI mice (*p* = 0.0006; Figure 2b). The three‐arm maze test (*p* = 0.699; Figure 2c) and the eight‐arm maze test (*p* = 0.646; Figure 2d) did not indicate any significant difference in working memory between the two groups. These results suggest that APP/IR‐dKI mice exhibit cognitive impairment mediated by nAChRα7 dysfunction.

### 2.3 Contribution of nicotinic acetylcholine α7 receptor to CBF regulation was reduced in APP/IR‐dKI mice

**FIGURE 3:**
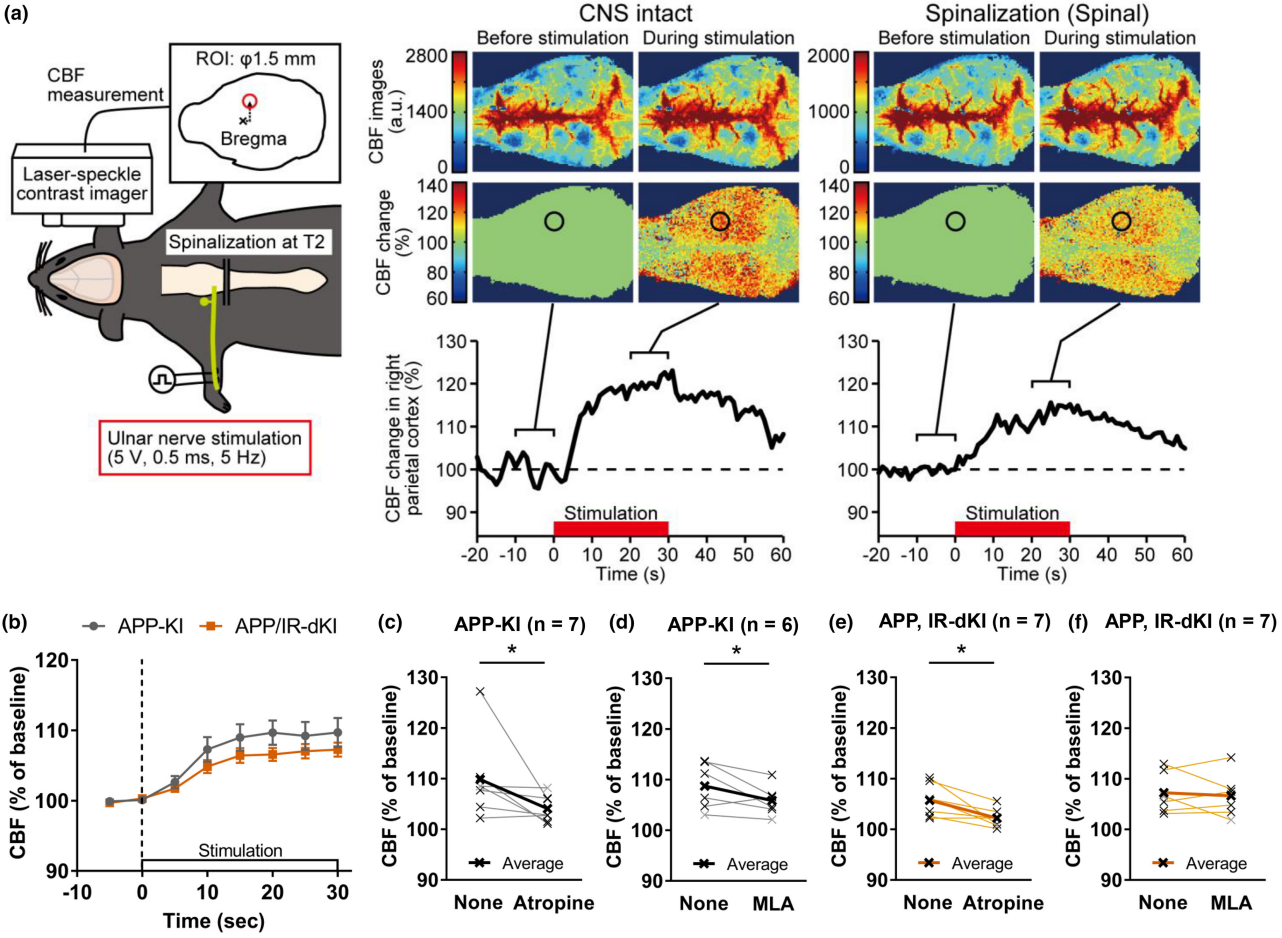
Contribution of nicotinic acetylcholine α7 receptor to cortical blood flow (CBF) regulation was reduced in APP/IR‐dKI mice. (a) Schematic illustration of electric stimulation of the ulnar nerve and CBF detection by laser speckle imaging CBF images before and during the ulnar nerve stimulation are presented. Each image is averaged for 10 s. The CBF image is expressed as a percentage change with respect to the image immediately before ulnar stimulation (100%). The time course of CBF changes in the right parietal cortex is presented. The location of CBF is indicated by a circle in the image, and the period of nerve stimulation is indicated by a thick horizontal bar. The left and right images and the graph are the results from the Wt mice under the intact condition on the central nervous system (CNS) and the condition after spinalization, respectively. (b) CBF responses to ulnar nerve stimulation under spinalization. There was no significance in both conditions (APP‐KI mice, *n* = 13; APP/IR‐dKI mice, *n* = 14). (c–f) CBF responses were compared before and 15 min after administering atropine (10 mg/kg) or MLA (5 mg/kg). Average CBFs from 10 to 20 s after the start of stimulation were compared (APP‐KI mice + atropine, *n* = 7; APP‐KI + MLA mice, *n* = 6; APP/IR‐dKI mice + atropine, *n* = 7; APP/IR‐dKI mice + MLA, *n* = 7). Although atropine decreased CBF response in both APP‐KI and APP/IR‐dKI mice, MLA decreased CBF response in APP‐KI mice but not in APP/IR‐dKI. Values represent the mean ± SEM *p < 0.05.

To evaluate the cholinergic function of APP/IR‐dKI mice, the CBF response to electrical stimulation of the ulnar nerve was monitored (Figure 3a). The CBF responses were triggered by the electrical stimulation in both APP‐KI and APP/IR‐dKI mice, without any difference between these two genotypes (Figure S2). The increase in CBF after electrical stimulation of the ulnar nerve was affected by elevated systemic blood pressure in mice that did not undergo spinalization (Inada et al., 1999; Sato et al., 1981). Thus, spinalization at thoracic spine T2 was performed in the mice to exclude this factor. A smaller CBF response was observed in spinalized wild‐type (Wt) mice compared to that before this operation (Figure S3). Similar to Wt mice, even after the spinalization, the CBF responses to the peripheral stimulation were observed in APP‐KI and APP/IR‐dKI mice (*p* > 0.999 at 5 s, *p* = 0.0214 at 10 s, *p* < 0.0001 at 15, 20, 25, 30 s after the stimulation compared with 0 s for APP‐KI; *p* > 0.999 at 5 s, *p* = 0.0236 at 10 s, *p* = 0.0001 at 15 s, and *p* < 0.0001 at 15, 20, 25, 30 s after the stimulation compared with 0 s for APP‐KI; Figure 3b). In this condition, there was no difference in CBF response to peripheral stimulation comparing between APP‐KI mice and APP/IR‐dKI mice (*p* = 0.552 at 5 s, *p* = 0.427 at 10 s, *p* = 0.382 at 15 s, *p* = 0.169 at 20 s, *p* = 0.519 at 25 s, *p* = 0.430 at 30 s after the stimulation; Figure 3b). To elucidate the cholinergic regulation on CBF in APP‐KI and APP/IR‐dKI, the pharmacological effects of the muscarinic receptor antagonist atropine and the nAChRα7 blocker MLA were analyzed. The CBF response in APP‐KI mice was decreased by both atropine (*p* = 0.0476; Figure 3c) and MLA (*p* = 0.0431; Figure 3d). In contrast, although atropine inhibited the CBF response in APP/IR‐dKI mice (*p* = 0.0108; Figure 3e), MLA did not affect the CBF response of this genotype (*p* = 0.311; Figure 3f). These results suggest that the contribution of nAChRα7 to the CBF response is smaller in APP/IR‐dKI mice than in APP‐KI mice.

### 2.4 Cortical gene expression related to neuronal activity and cholinergic transmission was reduced in APP/IR‐dKI mice

**FIGURE 4:**
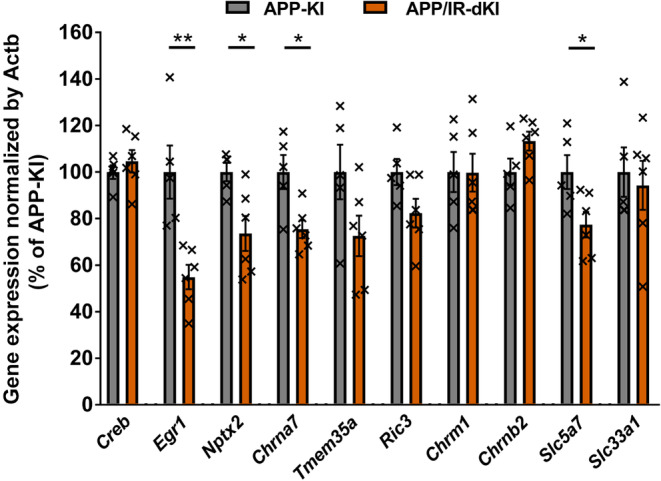
Cortical gene expression related to neuronal activity and cholinergic transmission was reduced in APP/IR‐dKI mice. Expression of genes related to neuronal activity and cholinergic function was evaluated by quantitative RT‐PCR (APP‐KI mice, *n* = 5; APP/IR‐dKI mice, *n* = 6). Values represent the mean ± SEM *p < 0.05, **p < 0.01.

To explore the mechanism of cognitive dysfunction and altered CBF responses, cortical gene expression related to neuronal activity and the cholinergic system was examined (Figure 4). Reduced expression of the immediate early genes, *Egr1* and *Nptx2*, and unaltered *Creb* gene expression were observed in APP/IR‐dKI mice compared with APP‐KI mice (*Creb*, *p* = 0.458; *Egr1*, *p* = 0.0041; *Nptx2*, *p* = 0.0170). Consistent with Figures 2 and 3, decreased expression of *Chrna7*, which encodes nAChRa7, but not of *Tmem35a* or *Ric3*, which encode regulatory proteins that localize nAChRa7 to the cell membrane (Gu et al., 2006; Williams et al., 2005), was observed in APP/IR‐dKI mice (*Chrna7*, *p* = 0.0120; *Tmem35a*, *p* = 0.0877; *Ric3*, *p* = 0.0670). The expression of genes encoding other cholinergic receptors, *Chrnm1* or *Chrnb2*, was not affected (*Chrnm1*, *p* = 0.984; *Chrnb2*, *p* = 0.0856). Additionally, the expression of *Slc5a7* mRNA, which encodes a choline transporter, was decreased in APP/IR‐dKI mice, whereas the expression of *Slc33a1* mRNA, which encodes an acetyl‐CoA transporter, was not altered (*Slc5a7*, *p* = 0.0329; *Slc33a1, p* = 0.714). These results suggest decreased neuronal activity and altered cholinergic regulation in the cortical regions of APP/IR‐dKI mice.

### 2.5 No acceleration of Aβ burden was observed in APP/IR‐dKI mice

**FIGURE 5:**
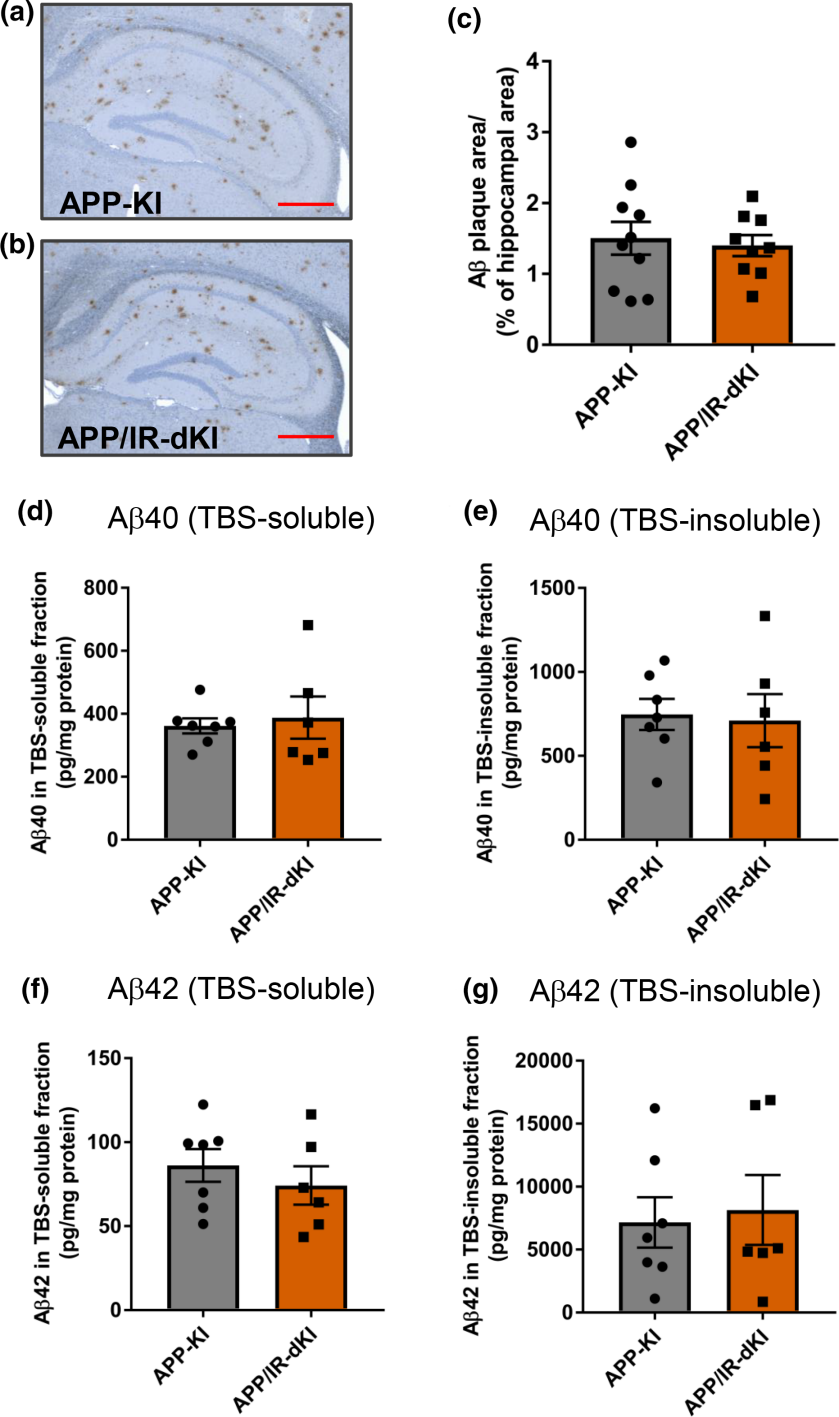
No acceleration of amyloid β (Aβ) burden was observed in APP/IR‐dKI mice. (a, b) Representative images of Aβ plaques in (a) APP‐KI and (b) APP/IR‐dKI mice. (c) Quantitative analysis was performed, and Aβ deposition was determined by calculating the ratio of the Aβ area to the hippocampal area (APP‐KI mice, *n* = 10; APP/IR‐dKI mice, *n* = 9). (d–g) The contents of Aβ40 and Aβ42 in the TBS‐soluble and insoluble fractions in the brain hemisphere were measured by ELISA (APP‐KI mice, *n* = 7; APP/IR‐dKI mice, *n* = 6). Values represent the mean ± SEM.

Finally, the Aβ burden was compared between APP‐KI and APP/IR‐dKI mice. Hippocampal Aβ deposition detected by immunohistochemical staining was not altered in APP/IR‐dKI mice compared with that in APP‐KI mice (*p* = 0.722; Figure 5a–c). Similarly, the amount of Aβ40 and Aβ42 in tris‐buffered saline (TBS)‐soluble and insoluble fractions measured by performing enzyme‐linked immunosorbent assay (ELISA) did not differ between APP‐KI mice and APP/IR‐dKI mice (Aβ40 in TBS‐soluble fraction, *p* = 0.704; Aβ40 in TBS‐insoluble fraction, *p* = 0.840; Aβ42 in TBS‐soluble fraction, *p* = 0.439; Aβ42 in TBS‐insoluble fraction, *p* = 0.773; Figure 5d–g). These results suggest that there was no acceleration of the Aβ burden in APP/IR‐dKI mice.

## 3 DISCUSSION

To clarify the effect of insulin resistance on the preclinical stage of AD, APP‐KI and APP/IR‐dKI mice were analyzed at an earlier age when the APP‐KI mice showed no severe cognitive dysfunction. IR‐KI mice, which harbor the P1195L mutation in the IR gene, exhibit features of insulin resistance such as glucose intolerance, insulin intolerance, and hyperinsulinemia, with normal blood glucose levels in fasting and fed states (Baba et al., 2005; Shimizu et al., 2011). These phenotypes were preserved in APP/IR‐dKI mice in this study (Figure 1). In addition, excessive temporal glucose elevation, probably due to delayed glucose uptake caused by insulin resistance, was observed in APP/IR‐dKI mice compared with APP‐KI mice, which indicates glucose fluctuation in the APP/IR‐dKI mice through daily maintenance by feeding normal rodent chow. Among the pathological factors of T2DM, insulin resistance and acute glucose fluctuations are associated with increased risk of AD and cognitive decline (Biessels et al., 2006; Ott et al., 1999; Zuin et al., 2021). Therefore, APP/IR‐dKI mice possess essential factors for T2DM related to AD pathology. At 4 months of age, APP‐KI mice begin to show cognitive decline according to the touchscreen‐based behavioral test, which can sensitively detect subtle cognitive changes (Saifullah et al., 2020). In the present study, APP‐KI mice did not show apparent cognitive impairment according to the novel object recognition and novel place preference tests, whose sensitivities are lower than touchscreen‐based test (Figure 2), while APP/IR‐dKI mice exhibited obvious cognitive dysfunction during the behavioral tests at the same age. In contrast, declining working memory was absent in 4‐month‐old APP/IR‐dKI mice. A detailed investigation of the cognitive properties would enable the evaluation of APP/IR‐dKI mice as a model for AD patients with T2DM.

The energy supplied by carbohydrates to neurons is largely regulated by astrocytes (Koepsell, 2020). Glucose that is taken up by astrocytes from the blood through an insulin‐independent glucose transporter, Glut1, is converted via glycolysis to lactate, which is consumed in neurons as energy (Dienel, 2017). Insulin signaling in the central nervous system has also been clarified. The insulin‐dependent glucose receptor Glut4, which is mainly expressed in astrocytes, contributes to cognitive function (Pearson‐Leary et al., 2018; Pearson‐Leary & McNay, 2016). Neuron‐specific IR‐knockout mice exhibit psychiatric alterations mediated by impaired dopamine turnover (Kleinridders et al., 2015). In our study, APP/IR‐dKI mice exhibit insulin resistance not only in the brain but also in the whole body. Further investigations are necessary to examine whether cognitive impairment mediated by cholinergic alterations observed in APP/IR‐dKI mice is caused by central insulin resistance.

Pharmacological and gene expression analyses revealed the dysfunction and downregulation of nAChRα7 (Figures 2, 3, 4). Reduced expression of nAChRα7 has been reported to impair cognitive functions, including long‐term memory (Curzon et al., 2006; Fernandes et al., 2006), consistent with our results. Interaction between glucose metabolism and nicotinic transmission has been suggested before (Duncan et al., 2019). The role of the cholinergic system, including both muscarinic and nicotinic receptors, in CBF regulation has been previously reported (Hotta, 2016; Lecrux et al., 2017). In this study, the contribution of nAChRα7 was different between APP‐KI and APP/IR‐dKI mice. Aβ plaques are deposited in the brains of both mice at the time of the CBF experiments. In a two‐photon imaging study, we recently found a smaller dilation response in arteries with Aβ plaques than in those without plaques (Watanabe et al., 2020). Microscale observation of cerebral blood vessels is necessary to reveal the synergistic effects of Aβ deposition and downregulation of nAChRα7 on CBF regulation. Considering the concept of neurovascular coupling and pointing out the close link between neuronal activity and local CBF responses (Iadecola, 2017), the mechanistical relationship between cognitive dysfunction and altered CBF responses should be investigated.

According to the gene expression analyses, the expression of neuronal activity markers (*Egr1* and *Nptx2*) and *Chrna7* was reduced in APP/IR‐dKI mice compared with APP‐KI mice (Figure 4). Insulin signaling in the brain affects neuronal activity (Suzuki et al., 2010). Expression of EGR1 was also reported to decrease in the frontal cortex of AD patients (Hu et al., 2019; Jin et al., 2023). Considering that EGR1 is involved in cholinergic function (Jin et al., 2023; Quirin‐Stricker et al., 1997), reduced expression of Egr1 in APP/IR‐dKI could reflect cholinergic malfunction in these mice. NPTX2, which expresses on GABAergic neurons inhibiting excitatory pyramidal neurons (Chang et al., 2010), was downregulated in the frontopolar cortex of AD patients (Xiao et al., 2017), which causes the excitatory/inhibitory imbalance of memory impairment (Xiao et al., 2017). In this study, since cortical expression of Nptx2 was reduced in APP/IR‐dKI compared with APP‐KI, the excitatory/inhibitory imbalance could be worsened. Considering the possibility that nAChRα7 downregulation contributes to cognitive dysfunction through insulin resistance, nAChRα7 could mediate the neuronal activity modulated by insulin signaling. Interestingly, APP/IR‐dKI mice exhibited not only a reduction in *Chrna7* expression but also a decreased tendency of *Ric3* and *Tmem35a* expression, which are involved in the stable function of nAChRα7 on the cell membrane (Gu et al., 2006; Williams et al., 2005). Insulin signaling could modulate these gene expressions via common mechanisms. Furthermore, the expression of the *Slc5a7*, which encodes transporter of choline and is a source of acetylcholine, was also reduced in APP/IR‐dKI mice. Choline transporters are also involved in cognitive processes (Paolone et al., 2013), and their activity is insulin‐sensitive (Fishwick & Rylett, 2015). These studies do not contradict our results.

APP/IR‐dKI mice did not show accelerated Aβ burden compared with APP‐KI mice (Figure 5), which suggests that cognitive dysfunction and altered CBF regulation are not a result of Aβ accumulation. Considering that the deposition of Aβ plaques is not necessarily associated with cognitive status in patients with AD (Esparza et al., 2013; Price & Morris, 1999) or in the rodent model of AD (Izuo et al., 2017, 2019), it is not surprising that no correlation was observed between Aβ load and cognitive dysfunction in this study. However, it is difficult to determine whether insulin resistance is related to Aβ accumulation, partly because the Arctic mutation, which is located on Aβ in APP‐KI mice, causes rapid aggregation (Murakami et al., 2003; Saito et al., 2014), possibly resulting in an underestimation of the possible effect of insulin resistance on Aβ accumulation. Among the Aβ clearance mechanisms, insulin and Aβ are substrates of the insulin‐degrading enzyme (Farris et al., 2003). Hyperinsulinemia in T2DM might competitively suppress Aβ degradation and thereby induce Aβ accumulation. However, the effect of T2DM on Aβ pathology remains controversial (Imamura et al., 2020; Ohara et al., 2011; Okabayashi et al., 2015; Sadrolashrafi et al., 2021; Takeda et al., 2010). Macauley et al. reported that a blood glucose clamp at higher hourly levels increased the levels of glucose and Aβ in the interstitial fluid of the hippocampus of AD model mice (Macauley et al., 2015). Glucose fluctuation observed in the present study could enhance Aβ production. The study using the mice generated by the crossbreeding with IR‐KI and AD model mice producing human WT‐Aβ would offer clear information on the role of insulin resistance in plaque pathology.

In mammals, insulin signaling is mediated by the insulin/IR and insulin‐like growth factor (IGF)‐1/IGF‐1 receptor (IGF‐1R) pathways. These two pathways originate from a single gene, *daf‐2*, in *Caenorhabditis elegans* (Kimura et al., 1997). Reduced expression of IGF‐1R in mice results in increased longevity (Holzenberger et al., 2003) and amelioration of AD (Gontier et al., 2015). Since the insulin/IR and IGF‐1/IGF‐1R pathways share the insulin receptor substrate family as downstream targets (Ochiai et al., 2021; Sesti et al., 2001; Tanokashira et al., 2019), conducting exclusive molecular analyses on insulin/IR signaling might be too complicated. Given that the phenotypes of APP/IR‐dKI mice in this study were opposite to those of IGF‐1R knockout mice, the exacerbated effect of insulin resistance on AD pathology could be derived from a molecular downstream of the insulin/IR pathway rather than of the IGF‐1/IGF‐1R pathway. Previously, downregulation of both the insulin/IR and IGF‐1/IGF‐1R pathways was observed in a brain autopsy of patients with AD (Talbot et al., 2012). Therefore, targeting the insulin/IR pathway‐specific cascade may be an effective strategy for the treatment of AD.

Currently, the development of AD therapeutics has been largely focused on antibody drugs. However, considering the high cost of treatment, it is important to prevent or delay the onset of AD in patients with risk factors such as T2DM. Therefore, interventions must be implemented during the preclinical stages of AD. In this study, APP/IR‐dKI mice that showed insulin resistance without persistent hyperglycemia exhibited an earlier onset of cognitive dysfunction. This model is expected to be utilized in the development of novel therapeutic strategies for AD.

## 4 METHODS

### 4.1 Animals

All experimental procedures were performed in accordance with specified guidelines for the care and use of laboratory animals and were approved by the Animal Care and Use Committee of Chiba University (approval number: 1‐282), the National Center for Geriatrics and Gerontology (approval number: 2‐24), the Tokyo Metropolitan Institute for Geriatrics and Gerontology (approval number: 18025‐2), the committee for recombinant DNA experiments at Chiba University (approval number: 28‐79), the National Center for Geriatrics and Gerontology (approval number: 2‐28), and the Tokyo Metropolitan Institute for Geriatrics and Gerontology (approval number: 188). Experimental animals were maintained at 24 ± 1°C and 55% ± 10% relative humidity under a 12 h light/dark cycle, with ad libitum access to standard rodent chow and water. In this study, APP‐KI, IR‐KI, and APP/IR‐dKI mice represented APP (homozygote)‐KI, IR (heterozygote)‐KI, and APP (homozygote)/IR (heterozygote)‐dKI mice, respectively. APP‐KI and APP/IR‐dKI mice were produced by breeding male APP (homozygote)/IR (heterozygote)‐dKI mice with female APP (homozygote)‐KI mice because female APP (homozygote)/IR (heterozygote)‐dKI mice frequently kill their newborn pups. Male mice were used in all experiments because female IR‐KI mice exhibit a weak insulin‐resistant phenotype because of the effect of estrogen (Baba et al., 2005). As originally reported, APP‐KI mice exhibit Aβ deposition at 2 months of age and cognitive impairment at 6 months of age (Saito et al., 2014; Sasaguri et al., 2017). In this study, metabolic analyses were performed at 3 months of age, and the other experiments were conducted at 4 months of age.

### 4.2 Metabolic tests

Blood glucose levels in the mice were determined using an automatic monitor (Glucocard; Arkray Inc.) applied to the blood from a cut on the tail. In the insulin tolerance tests, blood glucose levels were measured at 0, 10, 30, and 60 min after intraperitoneal injection of insulin (1 unit/kg) into the mice. In the glucose tolerance tests, blood glucose levels were measured at 0, 15, 30, and 60 min after the intraperitoneal injection of glucose (2 g/kg) into the mice that had undergone fasting overnight. The point at 0 min corresponds to the fasting blood glucose level. Insulin levels in the serum of fed mice were determined using an ELISA for mouse insulin (#27705; Immuno‐Biological Laboratories Inc., Fujioka, Japan).

### 4.3 Behavioral tests

#### 4.3.1 Novel object recognition test

The novel object recognition test was performed to evaluate long‐term memory as described in the previous study (schematic diagram of Figure 2a) (Chino et al., 2022). Before the test, mice were placed in an open box (45 × 45 × 15 cm) for 10 min over five consecutive days for habituation. On Day 6, the mice were placed facing two similar objects (P and P′) for 10 min over two successive days (acquisition phase) for 2 days. On Day 8, mice were placed facing object P and a novel object (Q) for 10 min (test phase). During the test phase, the number of times the mouse touched each object with its nose to explore it was counted. Exploratory preference was scored as the ratio of the number of times the novel object Q was touched with the nose to the total number of times both objects P and Q were touched with the nose. It was confirmed that the mice showed no significant preference for any of the objects before the test. Subsequently, nAChRα7 antagonist MLA (5 mg/kg) (TOCRIS Bioscience, Minneapolis, MN, USA) was administered intraperitoneally 15 min before the test. All mice that touched the objects less than five times with their noses were eliminated from the analysis.

#### 4.3.2 Novel place preference test

The novel place preference test was conducted to evaluate long‐term memory by following the revised procedure of the previous study (schematic diagram of Figure 2b) (Murakami et al., 2011). A Y‐maze test apparatus (Muromachi Kikai) made of 10 cm‐high gray plastic walls and consisting of three compartments (41.5 × 4 cm) connected with 4 × 4 cm passages was used for this experiment. The mice were placed at the end of one arm (Arm 1) of the maze and allowed to freely explore Arm 1 and the passage area for 5 min (training phase). The next day, the mice were placed at the end of Arm 1 and allowed to freely explore Arm 1, the newly opened arm (Arm 2) as a novel place, and the passage area for 10 min (test phase). Exploratory preference was scored as the ratio of time spent in Arm 2 to the total time spent in Arms 1 and 2. The time after the first entry of all four paws into Arm 2 was used for scoring. All mice with total number of entries less than five or those that showed massive incontinence were eliminated from the analysis.

#### 4.3.3 Three‐arm maze test

A three‐arm maze test was performed to evaluate working memory according to the previous study (schematic diagram of Figure 2c) (Maki et al., 2022). The Y‐maze test apparatus was also used in this experiment. The mice were placed at the end of one arm and allowed to explore all three arms freely for 5 min. Arm entry was recorded when all four paws entered the compartment. Alternation response was defined as entry into the arm that was not last selected for entry. The alternation response rate was calculated as the ratio of the number of alternations to the number of opportunities for choosing an arm to enter. All mice with a total entry number <5 were eliminated from the analysis.

#### 4.3.4 Eight‐arm maze test

The eight‐arm maze test was performed to evaluate working memory (schematic diagram of Figure 2d). A radial maze test apparatus made of 10 cm‐high gray plastic walls and consisting of eight compartments (35 × 7 cm) connected to a circular central region with a diameter of 20 cm was used for this experiment. A transparent cylinder with a diameter of 15 cm was placed at the center of the apparatus. Mice were placed in this cylinder and allowed to look out from the center. One minute later, the cylinder was removed, and the mice were allowed to freely explore the apparatus for 10 min. Arm entry was recorded when all four paws entered the compartment. Alternation response was defined as entry into the arm that was not selected in the last five opportunities for entry. For example, arm choice of “3” following arm entry of “8742” or “4” following “5261” are alternation responses, and arm choice of “4” following arm entry of “1734” or “5” following “6356” are not alternation responses. The alternation response rate was calculated as the ratio of the number of alternations to the number of opportunities to choose an arm to enter.

### 4.4 Measurement of CBF response to ulnar nerve stimulation

Animals were anesthetized with sevoflurane (4% for induction, 2.4%–3.1% for maintenance), and a sufficient depth of anesthesia was evaluated based on the loss of corneal and withdrawal reflexes. Body temperature was maintained at 37.5°C using a heating pad and lamp (ATB‐1100; Nihon Kohden). The left ulnar nerve was exposed and isolated from the surrounding connective tissues. The nerve was transected and ligated at the wrist using a thread, and its proximal side was placed on platinum‐iridium bipolar hook electrodes and covered with petroleum jelly. The ulnar nerve was electrically stimulated using a digital electrical stimulator (SEN‐7203; Nihon Kohden) and stimulus isolation unit (SS‐202J; Nihon Kohden) at 5 V and 5 Hz for 30 s with a pulse duration of 0.5 ms.

The head of each mouse was fixed to a stereotaxic instrument using ear bars (SR‐5 M‐S; Narishige). The skin of the scalp was excised, and the skull was exposed. The skull was kept intact, and liquid paraffin oil was used to prevent drying. CBF was measured using a laser speckle contrast imager consisting of an infrared semiconductor laser (wavelength of 785 nm) and a charge‐coupled device camera (Moor LFPI; Moor Instruments, Devon, UK). The device was set above the dorsal head of the mouse and adjusted to include the whole brain in the image. The field of view was approximately 242.5 mm^2^ (18.1 × 13.4 mm). One plane image consisted of 152 × 113 pixels with a pixel size of approximately 119 μm. Images were acquired at a rate of 25 fps with an exposure time of 4 ms. To quantify the regional CBF, blood flow data were averaged every 5 s and extracted by considering a region of interest (ROI) with a diameter of 1.5 mm (MoorFLPI Review V5.0, Moor Instruments). The ROI was on the right parietal cortex, ranging from 0 to 1.5 mm on the posterior side and 1.5 to 3 mm on the lateral side from the bregma). Spinalization at thoracic spine T2 was performed to exclude the factor of blood pressure and confirmed the loss of increase in CBF in response to tail pinching in all mice. Additionally, blood pressure was measured before and during ulnar nerve stimulation using a noninvasive blood pressure monitor in five mice (MK‐2000ST, Muromachi Kikai Co., Ltd.) and confirmed that systolic blood pressure did not differ before and during ulnar nerve stimulation in 5 mice (67 ± 4.9 vs. 67 ± 5.4 mmHg, respectively, *p* = 0.501). Either 10 mg/kg atropine (Sigma‐Aldrich Inc.) or 5 mg/kg MLA were administered intraperitoneally 15 min before the test. The CBF response was represented by the ratio to the baseline determined by the average for 10 s before the stimulation. The pharmacological effects on the CBF response to nerve stimulation were analyzed on an average from 10 to 20 s after initiating electric stimulation.

### 4.5 Brain sampling

The mice were administered anesthesia through intraperitoneal injection of three mixed anesthetics (0.75 mg/kg domitol, Orion Corp.; 4 mg/kg midazolam, SANDOZ Co., Ltd.; and 5 mg/kg butorphanol, Meiji Seika Pharma Co., Ltd.) and then perfused with ice‐cold phosphate‐buffered saline (PBS) to remove their blood. The brains or cortical tissues were collected and placed in TRIzol RNA Isolation Reagent (Thermo Fisher Scientific) for RNA extraction, fixed by soaking in 4% paraformaldehyde (Nacalai Tesque, Inc.) for immunohistochemical staining, or frozen in liquid nitrogen for ELISA.

### 4.6 Quantitative reverse transcription polymerase chain reaction

Tissues were homogenized using the TRIzol reagent. The total RNA was extracted according to the manufacturer's instructions. cDNA was synthesized using a ReverTra Ace qPCR RT Kit (TOYOBO CO. Ltd., Osaka, Japan). Total cDNA (100 ng) was used as the template for real‐time reverse transcription polymerase chain reaction (RT‐PCR) analysis. cDNA was quantified using an ABI Prism 7500 sequence‐detection system with the primers described in Table S1 and SYBR Green PCR Master Mix (Applied BioSystems), according to the manufacturer's instructions. The detector was programmed with the following PCR conditions: 40 cycles of denaturation at 95°C for 15 s each and 1 min of amplification at 60°C. All reactions were performed in duplicate, and *Actb* was used as the internal control. Relative differences in PCR results were calculated using the comparative cycle threshold method.

### 4.7 Immunohistochemistry

First, 5 μm‐thick coronal paraffin‐embedded sections were prepared from fixed brain hemispheres. After deparaffinization and hydration, the brain sections were subjected to antigen activation by autoclaving in a 10 mM citric acid solution (pH 6.0) at 121°C for 20 min. To inhibit endogenous peroxidase activity, the brain sections were soaked in methanol containing 0.1% H_2_O_2_ for 30 min. After washing the sections with ice‐cold PBS containing 0.02% Tween‐20 (PBST), blocking was performed using PBST containing 10% goat serum (Sigma‐Aldrich) for 30 min at room temperature. The first antibody to the N‐terminus of Aβ (0.5 μg/mL, clone 82E1, Immuno‐biological Laboratories) was diluted with blocking buffer and incubated with the sections overnight at 4°C. Subsequently, the sections were washed with PBST, and the secondary antibody, biotinylated mouse IgG (Vector Laboratories, Newark, CA, USA), was diluted with blocking buffer and applied to the sections for 1 h at room temperature. Immunological signals were enhanced using an avidin–biotin complex reaction kit (Vector Laboratories), including horseradish peroxidase‐linked avidin. To visualize the signals, the brain sections were treated with 3,3′‐diaminobenzidine (Dojindo Laboratories, Kumamoto, Japan) in TBS with 0.1% H_2_O_2_. For reference, the nuclei were stained with 4′, 6‐diamino‐2‐phenylindole (Dojindo Laboratories). After being dehydrated and soaked in xylene, brain sections were mounted with a coverslip and reagent (Merck Millipore, Burlington, MA, USA).

### 4.8 Aβ ELISA

Frozen brain hemispheres were homogenized in TBS with protease inhibitors (Complete mini, Roche, Basel, Switzerland), 0.7 μg/mL pepstatin A, and 1 mM phenylmethylsulphonyl fluoride. After centrifugation (55,000 × *g*, 30 min, 4°C) of the homogenate, the resulting supernatant was collected as the TBS‐soluble fraction. The pellet was suspended in 6 M guanidine‐HCl (Fujifilm, Osaka Japan) and centrifuged (55,000 × *g*, 30 min, 4°C); the supernatant was collected as the TBS‐insoluble fraction. The concentrations of Aβ40 and Aβ42 in the TBS‐soluble and insoluble fractions were measured by ELISA (27,718 and 27,719, respectively; Immuno‐Biological Laboratories) in accordance with the manufacturer's instructions.

### 4.9 Statistics

All data are presented as the mean ± standard error of the mean (SEM). After the evaluation of normality using the Shapiro–Wilk test, statistical analyses were performed. In Figures 1, 2, 4, 5, and Figure S1, for comparing two genotype groups, an unpaired Student's *t* test or Mann–Whitney test (two‐tailed) was performed in accordance with the data normality. For comparing four groups, a one‐way ANOVA followed by Bonferroni's test was used for each group as normality was confirmed. In Figure 3, Figures S2, and S3, the CBF response to the nerve stimulation over time was analyzed using repeated measures ANOVA followed by Bonferroni's post hoc test or Friedman's test followed by Dunn's multiple comparisons test. For comparing the CBF response between the genotypes, an unpaired Student's *t* test or Mann–Whitney test (one‐tailed) was used. For comparing the CBF response between the CNS intact and spinalized conditions, the paired Student's *t* test or Wilcoxon matched‐pairs signed rank test was used. Statistical significance was set at *p* < 0.05. All statistical analyses were performed using Prism version 7 (Graph Pad Software).

## AUTHOR CONTRIBUTIONS

NI, NW, YN, HH, and TS designed the research. NI and NW wrote the manuscript. NI, NW, and YN performed experiments. NI and NW analyzed the data. NI, NW, YN, TS, TCS, KY, HH, and TS discussed the hypothesis and interpreted the data. TS, TCS, and KY gave critical suggestions. NI, NW, YN, TS, TCS, KY, HH, and TS edited the article. NI, HH, and TS coordinated and directed the project.

## FUNDING INFORMATION

This study was supported by JSPS KAKENHI (Grant 15K19279, 20K16006, 23K07007 to NI), a Grant‐in‐Aid for JSPS Fellows (Grant 16J05570 to NI), and Research Funding for Longevity Sciences (19–50 to TS) from the National Center for Geriatrics and Gerontology.

## CONFLICT OF INTEREST STATEMENT

The authors declare no competing interests.

## Supporting information


Data S1.

## Data Availability

The datasets generated during the current study are available from the corresponding author on reasonable request.
