# Supplementary material for: Insulin resistance induces earlier initiation of cognitive dysfunction mediated by cholinergic deregulation in a mouse model of Alzheimer's disease
Source: Aging Cell. 2023 Oct 11;22(11):e13994. doi: 10.1111/acel.13994 (PMC10652326; doi:10.1111/acel.13994)
Supplement: Supplementary file 1 — Data S1. [file ACEL-22-e13994-s001.pdf]

Supplementary information

Insulin resistance induces earlier initiation of cognitive dysfunction in model mice of Alzheimer's disease mediated by cholinergic deregulation.

Naotaka Izuo, Nobuhiro Watanabe, Yoshihiro Noda,

Takashi Saito, Takaomi C. Saido, Koutaro Yokote,

Harumi Hotta, Takahiko Shimizu

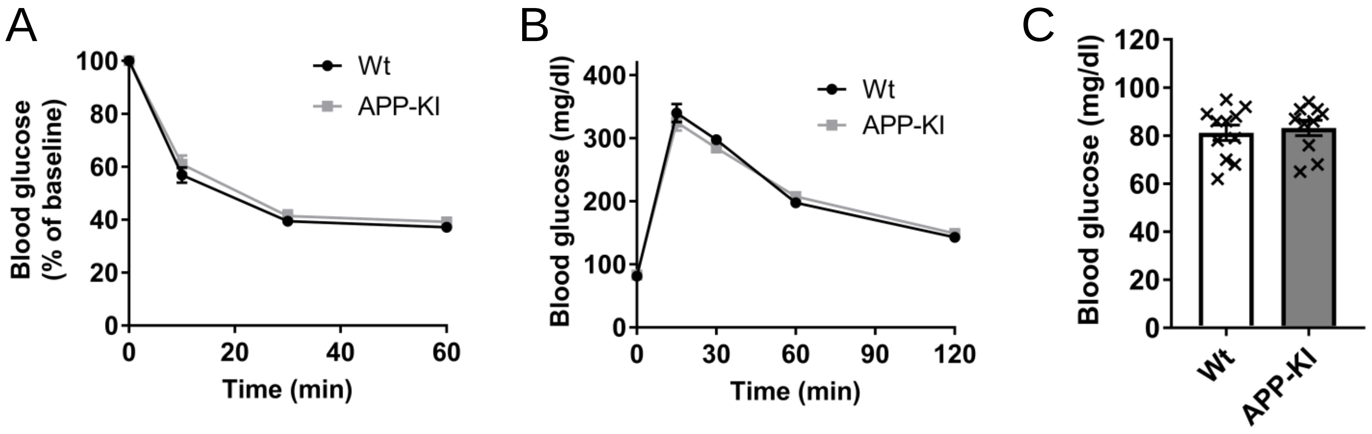

Supplementary figure 1. APP-KI mice exhibit no insulin resistance with normal basal blood glucose level.

No differences were observed between the (A) insulin tolerance test results (Wt, n = 11; APP-KI, n = 10; p = 0.370 at 10 min, p = 0.454 at 30 min, and p = 0.340 at 60 min, according to unpaired Student's t-test), (B) glucose tolerance test results (Wt, n = 11; APP-KI, n = 10; p = 0.662 at 0 min, p = 0.481 at 15 min, p = 0.151 at 30 min, p = 0.287 at 60 min, and p = 0.304 at 120 min), and (C) fasting blood glucose levels at 0 min, of Wt mice and APP-KI mice at 3 months of age. Values represent the mean ± S. E. M. Experiments were performed as described in the main text.

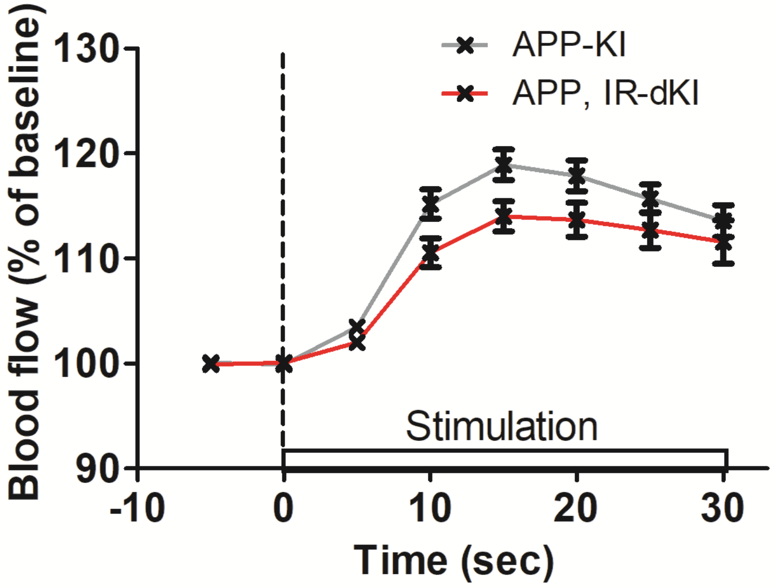

Supplementary figure 2. Comparison of CBF response to peripheral stimulation APP-KI and APP/IR-dKI in mice with the CNS intact.

The CBF responses were triggered by the electrical stimulation in both APP-KI and APP/IR-dKI mice, without difference between these two genotypes (APP-KI mice, n = 13; APP/IR-dKI, n = 14; p = 0.547 at 5 s, p = 0.0004 at 10 s, p < 0.0001 at 15, 20 s, p = 0.0002 at 25 s, p = 0.0054 at 30 s after the stimulation compared with 0 s analyzed by Friedman test followed by Dunn's multiple comparisons test for APP-KI; p = 0.0028 at 5 s, p < 0.0001 at 10, 15, 20, 25 s, p = 0.0006 at 30 s after the stimulation compared with 0 s, p < 0.0001, F_{Time} (13, 91) = 9.694, p < 0.0001 analyzed by ANOVA repeated measurements followed by Bonferroni's post hoc test for APP/IR-dKI; p = 0.524 at 5 s, p = 0.198 at 10 s, p = 0.264 at 15 s, p = 0.562 at 20 s, p > 0.999 at 25, 30 s after the stimulation compared with 0 s analyzed by unpaired Student's t-test or Mann Whitney test on each time point for comparing APP-KI and APP/IR-dKI mice). Values represent the mean ± S. E. M. Experiments were performed as described in the main text.

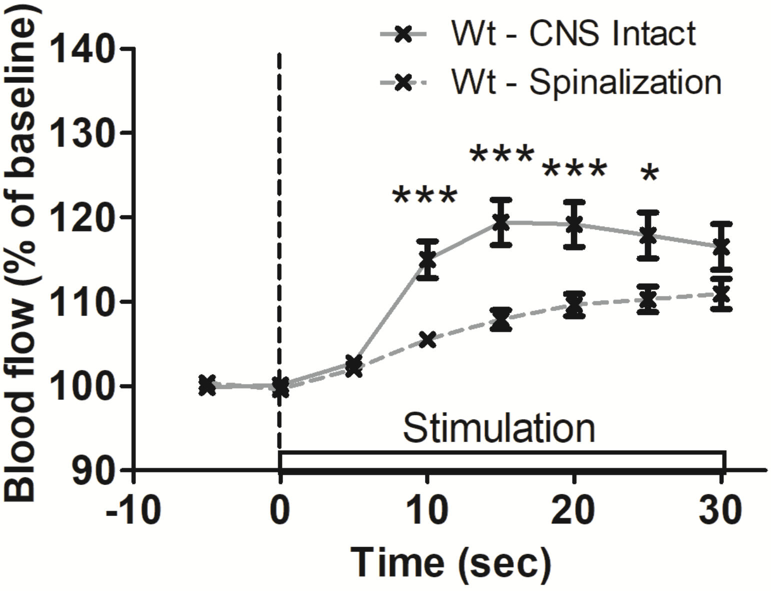

Supplementary figure 3. Comparison of CBF response to peripheral stimulation with or without spinalization in mice.
Electrical stimulation to peripheral nerve CBF increases in both CNS intact and spinalization conditions (n = 13, $p < 0.999$ at 5 s, $p = 0.0214$ at 10 s, $p < 0.0001$ at 15, 20, 25 s, $p = 0.0095$ at 30 s after the stimulation compared with 0 s analyzed by Friedman test followed by Dunn's multiple comparisons test for CNS intact condition; $p = 0.765$ at 5 s, $p = 0.0276$ at 10 s, $p = 0.0003$ at 15 s, $p < 0.0001$ at 20, 25, 30 s after the stimulation compared with 0 s analyzed by Friedman test followed by Dunn's multiple comparisons test for spinalization condition). Spinalization resulted in decreased CBF response to peripheral electric stimulation compared to intact Wt mice ($p < 0.999$ at 5 s, $p = 0.0012$ at 10 s, $p = 0.0006$ at 15 s, $p = 0.0030$ at 20 s, $p = 0.030$ at 25 s, $p = 0.134$ at 30 s after the onset of the stimulation, analyzed by paired Student's t-test or Wilcoxon matched-pairs signed rank test on each time point). Values represent the mean ± S. E. M. Experiments were performed as described in the main text.

Target	Sequence (5' to 3')	
Creb	Forward	ATCTGGAGCAGACAACCAGC
	Reverse	TGAGCTGCTGGCATGGATAC
Egr1	Forward	ACCTGACCACAGAGTCCTTTTC
	Reverse	GTCGGAGGATTGGTCATGCT
Nptx2	Forward	ATCAACGACAAGGTCGCACA
	Reverse	AACTGGCTGAGCTCTCCAAC
Chrna7	Forward	CGTGCCCTTGATAGCACAGTA
	Reverse	TTCATGCGCAGAAACCATGC
Teme35a	Forward	CCTACAGTGAAATGAAACGTGCT
	Reverse	GCGTTTGAGAGGATCACCCA
Ric3	Forward	GTACTCCACGGTGCAGAGAG
	Reverse	GTCCAATTTTCCTTCCGGGC
Chrm1	Forward	GGGCACACTCCAGGACTTAG
	Reverse	AGGGCATCACCATGGGACC
Chrnb2	Forward	GGCTGTGTTCAGGGGTTTTG
	Reverse	AGCGGGAAGGATCCAAGAGA
Slc5a7	Forward	GCAGCTTTTGGGTGCCTG
	Reverse	TGTGGAAGCTCCAATAGCTCC
Slc33a1	Forward	CTCAACCCAGGGGAATCGTT
	Reverse	CAGAAGGGCTACTAATGTTGTTGT
Actb	Forward	CGATGCCCTGAGGCTCTTTTC
	Reverse	GTTGGCATAGAGGTCTTTACGGATG

Supplementary table. Primers used for the experiments in quantitative RT-PCR.
